# 2,9-Dimethyl-1,10-phenanthrolin-1-ium 2,4,5-tri­carb­oxy­benzoate monohydrate

**DOI:** 10.1107/S1600536813030857

**Published:** 2013-11-16

**Authors:** Kai-Long Zhong

**Affiliations:** a Department of Applied Chemistry, Nanjing College of Chemical Technology, Nanjing 210048, People’s Republic of China

## Abstract

In the preparation of the title hydrated salt, C_14_H_13_N_2_^+^·C_10_H_5_O_8_^−^·H_2_O, a proton has been transfered to the 2,9-dimethyl-1,10-phenanthrolinium cation, forming a 2,4,5-tri­carb­oxy­benzoate anion. In the anion, the mean planes of the protonated carboxyl­ate groups form dihedral angles of 11.0 (5), 4.4 (5) and 80.3 (4)° with the benzene ring to which they are attached. The mean plane of the deprotonated carboxyl­ate group forms a dihedral angle of 10.6 (5)° with the benzene ring. In the crystal, the anions are involved in carb­oxy­lic acid O—H⋯O_carbox­yl_ hydrogen bonds, generating a two-dimensional network parallel to (001) containing *R*_4_^4^(28) and *R*_4_^4^(32) motifs. The 2,9-dimethyl-1,10-phenanthrolinium cations and water mol­ecules reside between the anion layers and are connected to the anions *via* N—H⋯O_water_ and O_water_—H⋯O_carbox­yl_ hydrogen bonds. An intra­molecular O—H⋯O hydrogen bond is also observed in the anion.

## Related literature

For related structures, see: Adams & Ramdas (1978 ▶); Mrvos-Sermek *et al.* (1996) ▶; Sun *et al.* (2002*a* ▶,*b* ▶); Zhu *et al.* (2002 ▶); Li *et al.* (2003 ▶; 2006 ▶); Oscar *et al.* (2008 ▶). For background to mol­ecular recognition and supra­molecular chemistry, see: Batten & Robson (1998 ▶); Juan *et al.* (2002 ▶); Qiu *et al.* (2008 ▶). For hydrogen-bond graph-set notation, see: Bernstein *et al.* (1995 ▶).
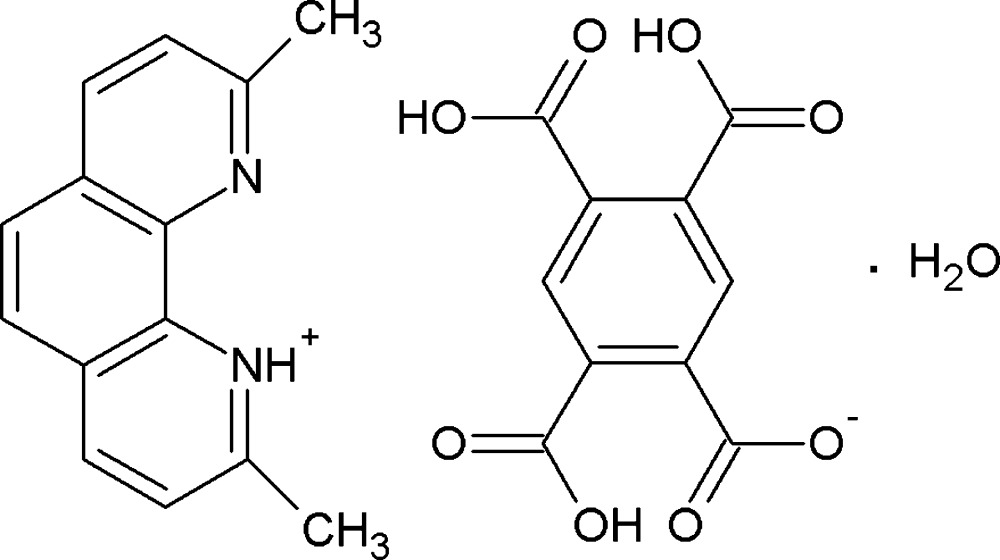


## Experimental

### 

#### Crystal data

C_14_H_13_N_2_^+^·C_10_H_5_O_8_^−^·H_2_O
*M* *_r_* = 480.42
Orthorhombic, 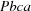

*a* = 7.1135 (8) Å
*b* = 19.4512 (11) Å
*c* = 30.800 (2) Å
*V* = 4261.7 (6) Å^3^
*Z* = 8
Mo *K*α radiation
μ = 0.12 mm^−1^
*T* = 223 K
0.35 × 0.20 × 0.15 mm

#### Data collection

Rigaku Mercury CCD diffractometer
Absorption correction: multi-scan (*REQAB*; Jacobson, 1998 ▶) *T*_min_ = 0.468, *T*_max_ = 1.000
19580 measured reflections
4346 independent reflections
2278 reflections with *I* > 2σ(*I*)
*R*_int_ = 0.173

#### Refinement

*R*[*F* ^2^ > 2σ(*F* ^2^)] = 0.088
*wR*(*F* ^2^) = 0.272
*S* = 1.00
4346 reflections
316 parameters
3 restraints
H-atom parameters constrained
Δρ_max_ = 0.37 e Å^−3^
Δρ_min_ = −0.40 e Å^−3^

### 

Data collection: *CrystalClear* (Rigaku, 2007 ▶); cell refinement: *CrystalClear*; data reduction: *CrystalClear*; program(s) used to solve structure: *SHELXS97* (Sheldrick, 2008 ▶); program(s) used to refine structure: *SHELXL97* (Sheldrick, 2008 ▶); molecular graphics: *SHELXTL* (Sheldrick, 2008 ▶), *PLATON* (Spek, 2009 ▶) and *Mercury* (Macrae *et al.*, 2008 ▶); software used to prepare material for publication: *SHELXTL*.

## Supplementary Material

Crystal structure: contains datablock(s) global, I. DOI: 10.1107/S1600536813030857/lh5664sup1.cif

Structure factors: contains datablock(s) I. DOI: 10.1107/S1600536813030857/lh5664Isup2.hkl

Supplementary material file. DOI: 10.1107/S1600536813030857/lh5664Isup3.cml

Additional supplementary materials:  crystallographic information; 3D view; checkCIF report

## Figures and Tables

**Table 1:** Hydrogen-bond geometry (Å, °)

*D*—H⋯*A*	*D*—H	H⋯*A*	*D*⋯*A*	*D*—H⋯*A*
O2—H2⋯O3	0.82	1.58	2.395 (4)	171
O5—H5⋯O1^i^	0.82	1.86	2.671 (3)	172
O8—H8⋯O3^ii^	0.82	1.82	2.645 (4)	178
N1—H1*A*⋯O1*W* ^iii^	0.86	1.92	2.738 (4)	160
O1*W*—H1*WA*⋯O4^ii^	0.82	1.92	2.735 (4)	171
O1*W*—H1*WB*⋯O7^iv^	0.82	2.11	2.873 (4)	155

Symmetry codes: (i) 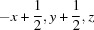; (ii) 
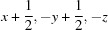
; (iii) 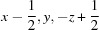; (iv) 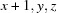.

**Fig. 1.**
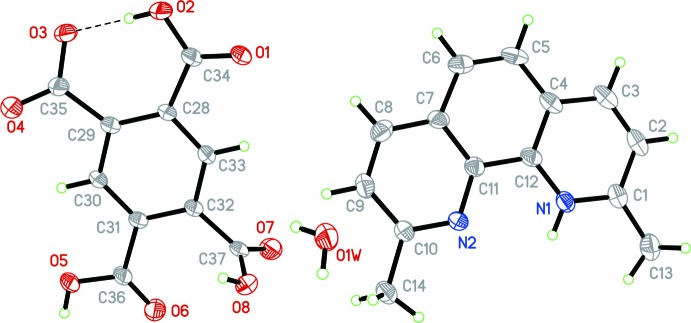
Figure 1. The asymmetric unit of (I), showing displacement ellipsoids drawn at the 30% probability level. An intramolecular hydrogen bond is shown as a dashed line.

**Fig. 2.**
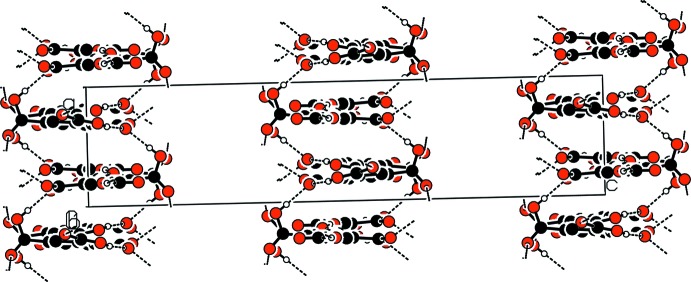
Figure 2. Part of the crystal structure of (I) showing layers parallel to (0 0 1). Hydrogen bonds are represented by dashed lines. The 2,9-dimethyl-1,10-phenanthrolinium cations and the water molecules have been omitted for clarity.

**Fig. 3.**
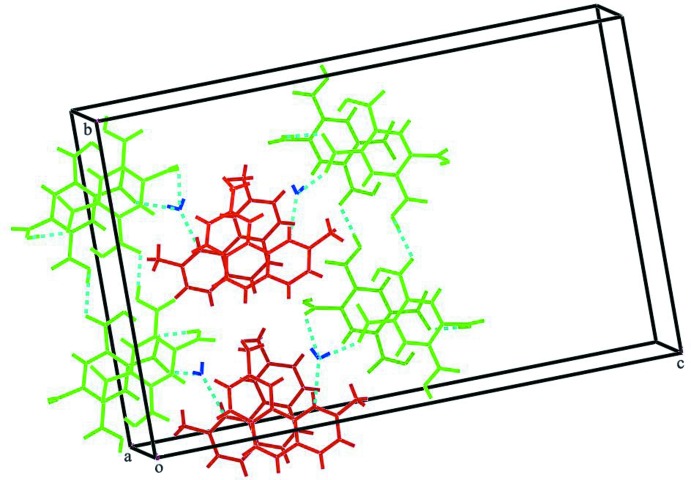
Figure 3. Part of the crystal structure showing hydrogen bonds involving the cations (red) and water molecules (blue). The hydrogen bond donor···acceptor distances are shown as dashed lines.

## supplementary crystallographic information

### 1. Comment

It is well established that hydrogen bonds play vital roles in molecular recognition and supramolecular chemistry (Batten & Robson 1998; Juan *et al.*, 2002; Qiu *et al.*, 2008), owing to their moderately directional intermolecular interaction which can effectively control short-range packing. 1,2,4,5-Benzenetetracarboxylic acid (Li *et al.*, 2003; Oscar *et al.*, 2008) has been widely applied in constructing interesting supramolecular networks because it acts not only as hydrogen bond acceptor but also as a hydrogen bond donor, depending upon the number of deprotonated carboxylate groups. Some proton-transfer compounds of 1,2,4,5-benzenetetracarboxylic acid with nitrogen Lewis bases, such as guanidinium pyromellitate trihydrate monoperhydrate (Adams & Ramdas, 1978), 2,2'-bipyridinium hemi[1,2,4,5- benzenetetracarboxylate(2-)] hemi(1,2,4,5-benzenetetracarboxylic acid) (Mrvos-Sermek *et al.*, 1996), guanidinuium 2,9-dimethyl-1,10-phenanthrolinium trihydrogen-1,2,3,4-teracarboxybenzoate monohydrate (Sun *et al.*,, 2002*a*), imidazolium trihydrogen-1,2,4,5-benzenetetracarboxylate (Sun *et al.*, 2002*b*), 6,21-diaza-3,9,18,24-tetraazoniatricyclo[22.2.2.2^11,14^] triaconta-11,13,24,26 (1),27,29-hexaene benzene-1,2,4,5-tetracarboxylate(4-) hexahydrate (Zhu *et al.*, 2002) and ethylenediammonium bis(trihydrogen-1,2,4,5-benzenetetracarboxylate) dehydrate (Li *et al.*, 2006) have been synthesized and reported. The title compound (I) was obtained unintentionally during an attempt to synthesize mixed-ligand transition metal complexes with 1,2,4,5-benzenetetracarboxylic acid and 2,9-dimethyl-1,10-phenanthroline ligands *via* a solvo thermal reaction. Its crystal structure is reported herein.

The asymmetric unit of (I) (Fig. 1) is comprised of a 2,9-dimethyl-1,10-phenanthrolinium cation, a trihydrogen-1,2,4,5-benzenetetracarboxylate anion and a solvent water molecule. The proton transfer from a carboxyl group to the ring N atom (N1) of the 2,9-dimethyl-1,10-phenanthroline is manifested in an increased internal angle [C1—N1—C12 = 123.7 (3)°] of the pyridine ring, compared with that for atom N2 of the pyridine ring [C10—N2—C11 = 117.8 (3)°]. The protonation diminishes the steric effect of the lone pair of electrons and is responsible for the slightly increased C1—N1—C12 angle. In the anion, the mean-planes of the protonated carboxylate groups form didedral angles of 11.0 (5) ° for C34/O1/O2, 4.4 (5)° for C36/O5/O6 and 80.3 (4) ° for C37/O7/O8, with the benzene ring to which they are attached. The mean-plane of the deprotonated carboxylate group C35/O4/O4 forms a dihedral angle of 10.6 (5) ° with the benzene ring. Symmetry-related trihydrogen-1,2,4,5-benzenetetracarboxylate anions interact *via* O5—H5···O1^i^ and O8—H8···O3^ii^ hydrogen bonds, forming a two-dimensional supramolecular layer parallel to (001), which involves *R*_4_^4^(28) and *R*_4_^4^(32) motifs (Bernstein *et al.*, 1995) (Fig. 2 & Table 1). The 2,9-dimethyl-1,10-phenanthrolinium cations are linked to the water molecules (O1W) through N1—H1A···O1W^iii^ hydrogen bonds and reside between the two-dimensional supramolecular layers and are connected to the layers *via* O1W—H1WA···O4^ii^ and O1W—H1WB···O7^iv^ hydrogen bonds (Fig. 3 & Table 1).

### 2. Experimental

2,9-Dimethyl-1,10-phenanthroline (0.1 mmol), 1,2,4,5-benzenetetracarboxylic acid (0.1 mmol), ZnSO_4_·7H_2_O (0.1 mmol) and 2.0 ml water were mixed and placed in a thick Pyrex tube, which was sealed and heated to 383 K for 72 h, whereupon colorless block-shaped crystals of (I) were obtained.

### 3. Refinement

The H atoms bonded to C atoms were were positioned geometrically and allowed to ride on their parent atoms, with C—H = 0.93, 0.96 Å; *U*_iso_(H) = 1.2*U*_eq_(C) or 1.5*U*_eq_(C_methyl_)). The H atoms bound to N and O were placed in calculated positions with N—H = 0.86 Å, O—H = 0.82 Å and refined with *U*_iso_(H) = 1.2*U*_eq_(N) and *U*_iso_(H) = 1.5*U*_eq_(O). The precision of the structure is slightly lower than normal as reflected in the R-factors. We attempted to use serveral crystals but the crystal used to collect the data herein was the best quality avaiable.

### Crystal data

**Table:**

C_14_H_13_N_2_^+^·C_10_H_5_O_8_^−^·H_2_O	*F*(000) = 2000
*M**_r_* = 480.42	*D*_x_ = 1.498 Mg m^−^^3^
Orthorhombic, *P**b**c**a*	Mo *K*α radiation, λ = 0.71073 Å
Hall symbol: -P 2ac 2ab	Cell parameters from 2016 reflections
*a* = 7.1135 (8) Å	θ = 3.4–26.0°
*b* = 19.4512 (11) Å	µ = 0.12 mm^−^^1^
*c* = 30.800 (2) Å	*T* = 223 K
*V* = 4261.7 (6) Å^3^	Block, colourless
*Z* = 8	0.35 × 0.20 × 0.15 mm

### Data collection

**Table:**

Rigaku Mercury CCD diffractometer	4346 independent reflections
Radiation source: fine-focus sealed tube	2278 reflections with *I* > 2σ(*I*)
Graphite Monochromator monochromator	*R*_int_ = 0.173
Detector resolution: 28.5714 pixels mm^-1^	θ_max_ = 26.4°, θ_min_ = 2.9°
ω scans	*h* = −8→7
Absorption correction: multi-scan (*REQAB*; Jacobson, 1998)	*k* = −24→23
*T*_min_ = 0.468, *T*_max_ = 1.000	*l* = −38→30
19580 measured reflections	

### Refinement

**Table:**

Refinement on *F*^2^	Primary atom site location: structure-invariant direct methods
Least-squares matrix: full	Secondary atom site location: difference Fourier map
*R*[*F*^2^ > 2σ(*F*^2^)] = 0.088	Hydrogen site location: inferred from neighbouring sites
*wR*(*F*^2^) = 0.272	H-atom parameters constrained
*S* = 1.00	*w* = 1/[σ^2^(*F*_o_^2^) + (0.1341*P*)^2^] where *P* = (*F*_o_^2^ + 2*F*_c_^2^)/3
4346 reflections	(Δ/σ)_max_ < 0.001
316 parameters	Δρ_max_ = 0.37 e Å^−^^3^
3 restraints	Δρ_min_ = −0.40 e Å^−^^3^

### Special details

**Table:**

Geometry. All e.s.d.'s (except the e.s.d. in the dihedral angle between two l.s. planes) are estimated using the full covariance matrix. The cell e.s.d.'s are taken into account individually in the estimation of e.s.d.'s in distances, angles and torsion angles; correlations between e.s.d.'s in cell parameters are only used when they are defined by crystal symmetry. An approximate (isotropic) treatment of cell e.s.d.'s is used for estimating e.s.d.'s involving l.s. planes.
Refinement. Refinement of *F*^2^ against ALL reflections. The weighted *R*-factor *wR* and goodness of fit *S* are based on *F*^2^, conventional *R*-factors *R* are based on *F*, with *F* set to zero for negative *F*^2^. The threshold expression of *F*^2^ > σ(*F*^2^) is used only for calculating *R*-factors(gt) *etc*. and is not relevant to the choice of reflections for refinement. *R*-factors based on *F*^2^ are statistically about twice as large as those based on *F*, and *R*- factors based on ALL data will be even larger.

### Fractional atomic coordinates and isotropic or equivalent isotropic displacement parameters (Å^2^)

**Table:**

	*x*	*y*	*z*	*U*_iso_*/*U*_eq_	
O1	0.2749 (5)	0.08753 (12)	0.04898 (9)	0.0620 (10)	
O1W	0.9045 (6)	0.17263 (15)	0.15048 (10)	0.0773 (11)	
H1WA	0.8321	0.1863	0.1316	0.116*	
H1WB	0.9732	0.2039	0.1586	0.116*	
O2	0.1964 (5)	0.09431 (13)	−0.01923 (9)	0.0687 (11)	
H2	0.1669	0.1239	−0.0369	0.103*	
O3	0.1453 (4)	0.18081 (12)	−0.07332 (9)	0.0514 (8)	
O4	0.1828 (5)	0.29265 (13)	−0.08131 (10)	0.0607 (9)	
O5	0.1946 (5)	0.45577 (12)	0.02561 (9)	0.0710 (11)	
H5	0.1941	0.4958	0.0340	0.106*	
O6	0.1961 (6)	0.43455 (14)	0.09547 (10)	0.0814 (12)	
O7	0.1129 (5)	0.29909 (14)	0.15299 (9)	0.0570 (9)	
O8	0.4094 (5)	0.32175 (13)	0.13965 (9)	0.0526 (8)	
H8	0.4812	0.3203	0.1189	0.079*	
N1	0.3348 (5)	0.04067 (14)	0.32309 (11)	0.0418 (8)	
H1A	0.3756	0.0820	0.3261	0.050*	
N2	0.4269 (5)	0.12165 (13)	0.25335 (10)	0.0387 (8)	
C1	0.2968 (6)	0.0044 (2)	0.35906 (14)	0.0505 (11)	
C2	0.2298 (6)	−0.0628 (2)	0.35368 (16)	0.0565 (12)	
H2B	0.2018	−0.0892	0.3780	0.068*	
C3	0.2051 (6)	−0.0901 (2)	0.31345 (16)	0.0540 (12)	
H3A	0.1608	−0.1348	0.3107	0.065*	
C4	0.2457 (6)	−0.05151 (18)	0.27592 (15)	0.0464 (10)	
C5	0.2205 (6)	−0.07699 (19)	0.23280 (16)	0.0510 (11)	
H5A	0.1792	−0.1218	0.2285	0.061*	
C6	0.2559 (6)	−0.0366 (2)	0.19861 (16)	0.0498 (11)	
H6A	0.2354	−0.0539	0.1709	0.060*	
C7	0.3240 (6)	0.03194 (17)	0.20314 (13)	0.0403 (9)	
C8	0.3644 (6)	0.0762 (2)	0.16851 (14)	0.0488 (10)	
H8A	0.3421	0.0621	0.1401	0.059*	
C9	0.4360 (6)	0.13951 (19)	0.17649 (13)	0.0473 (10)	
H9A	0.4648	0.1686	0.1535	0.057*	
C10	0.4672 (6)	0.16161 (16)	0.21950 (12)	0.0416 (10)	
C11	0.3565 (5)	0.05828 (17)	0.24523 (12)	0.0382 (9)	
C12	0.3122 (5)	0.01557 (17)	0.28188 (13)	0.0397 (10)	
C13	0.3258 (8)	0.0376 (2)	0.40228 (16)	0.0716 (15)	
H13A	0.3728	0.0834	0.3982	0.107*	
H13B	0.4149	0.0113	0.4188	0.107*	
H13C	0.2084	0.0394	0.4176	0.107*	
C14	0.5500 (6)	0.23092 (17)	0.22864 (14)	0.0505 (11)	
H14A	0.5598	0.2375	0.2595	0.076*	
H14B	0.4706	0.2659	0.2165	0.076*	
H14C	0.6728	0.2338	0.2158	0.076*	
C28	0.2079 (5)	0.20013 (15)	0.02367 (12)	0.0357 (9)	
C29	0.1912 (5)	0.25062 (16)	−0.00893 (13)	0.0350 (9)	
C30	0.1867 (5)	0.31977 (17)	0.00395 (12)	0.0378 (9)	
H30A	0.1756	0.3535	−0.0173	0.045*	
C31	0.1979 (5)	0.33993 (16)	0.04710 (12)	0.0376 (9)	
C32	0.2144 (6)	0.28974 (16)	0.07952 (12)	0.0389 (10)	
C33	0.2186 (6)	0.22157 (17)	0.06673 (12)	0.0394 (9)	
H33A	0.2291	0.1881	0.0881	0.047*	
C34	0.2288 (7)	0.12280 (18)	0.01805 (14)	0.0471 (11)	
C35	0.1756 (6)	0.24134 (18)	−0.05812 (13)	0.0411 (10)	
C36	0.1967 (6)	0.41454 (17)	0.05910 (13)	0.0457 (11)	
C37	0.2376 (7)	0.30587 (16)	0.12675 (13)	0.0428 (10)	

### Atomic displacement parameters (Å^2^)

**Table:**

	*U*^11^	*U*^22^	*U*^33^	*U*^12^	*U*^13^	*U*^23^
O1	0.119 (3)	0.0212 (13)	0.0460 (18)	0.0077 (14)	−0.0028 (17)	−0.0004 (12)
O1W	0.107 (3)	0.066 (2)	0.058 (2)	−0.0367 (18)	−0.023 (2)	0.0200 (15)
O2	0.131 (3)	0.0301 (14)	0.0452 (18)	0.0046 (16)	−0.0161 (18)	−0.0095 (13)
O3	0.077 (2)	0.0359 (14)	0.0416 (17)	0.0044 (13)	−0.0108 (15)	−0.0086 (12)
O4	0.095 (3)	0.0408 (16)	0.0464 (18)	−0.0027 (14)	−0.0044 (17)	0.0012 (14)
O5	0.149 (4)	0.0180 (13)	0.0458 (19)	−0.0050 (15)	−0.0022 (19)	0.0033 (12)
O6	0.171 (4)	0.0306 (15)	0.0427 (19)	0.0046 (17)	0.000 (2)	−0.0069 (14)
O7	0.075 (2)	0.0492 (17)	0.0465 (18)	−0.0069 (14)	0.0186 (17)	−0.0076 (13)
O8	0.072 (2)	0.0489 (15)	0.0371 (16)	−0.0054 (14)	−0.0002 (15)	−0.0068 (12)
N1	0.054 (2)	0.0278 (15)	0.044 (2)	0.0022 (14)	0.0008 (16)	0.0035 (14)
N2	0.048 (2)	0.0261 (14)	0.0421 (19)	0.0036 (13)	−0.0007 (16)	0.0003 (13)
C1	0.057 (3)	0.042 (2)	0.053 (3)	0.0034 (19)	−0.001 (2)	0.0130 (19)
C2	0.059 (3)	0.040 (2)	0.071 (3)	0.005 (2)	0.006 (3)	0.022 (2)
C3	0.052 (3)	0.033 (2)	0.078 (3)	0.0028 (18)	0.009 (2)	0.009 (2)
C4	0.043 (2)	0.0283 (18)	0.068 (3)	0.0023 (16)	0.001 (2)	0.0029 (19)
C5	0.046 (3)	0.030 (2)	0.077 (3)	−0.0055 (17)	−0.003 (2)	−0.014 (2)
C6	0.051 (3)	0.043 (2)	0.056 (3)	0.0024 (19)	−0.001 (2)	−0.016 (2)
C7	0.045 (2)	0.0305 (19)	0.045 (2)	0.0050 (15)	−0.0001 (19)	−0.0067 (17)
C8	0.054 (3)	0.053 (2)	0.039 (2)	0.008 (2)	−0.002 (2)	−0.0088 (19)
C9	0.056 (3)	0.043 (2)	0.044 (2)	0.0074 (19)	0.005 (2)	0.0094 (18)
C10	0.049 (3)	0.0318 (17)	0.044 (2)	0.0032 (16)	−0.0029 (19)	0.0021 (16)
C11	0.042 (2)	0.0335 (18)	0.039 (2)	0.0060 (16)	0.0018 (18)	−0.0017 (16)
C12	0.040 (2)	0.0285 (18)	0.051 (3)	0.0043 (15)	−0.0003 (19)	−0.0022 (17)
C13	0.109 (5)	0.058 (3)	0.048 (3)	0.000 (3)	−0.002 (3)	0.015 (2)
C14	0.066 (3)	0.034 (2)	0.052 (3)	−0.0008 (19)	0.004 (2)	0.0064 (18)
C28	0.047 (2)	0.0222 (17)	0.038 (2)	−0.0023 (14)	−0.0021 (17)	−0.0018 (15)
C29	0.041 (2)	0.0259 (17)	0.038 (2)	−0.0004 (15)	0.0001 (17)	−0.0025 (15)
C30	0.051 (3)	0.0236 (17)	0.039 (2)	−0.0029 (16)	−0.0039 (18)	0.0019 (15)
C31	0.051 (3)	0.0217 (16)	0.040 (2)	0.0003 (15)	0.0012 (18)	−0.0030 (15)
C32	0.057 (3)	0.0230 (17)	0.037 (2)	−0.0001 (15)	0.0026 (18)	−0.0014 (15)
C33	0.056 (3)	0.0260 (17)	0.036 (2)	−0.0019 (16)	0.0024 (19)	0.0029 (15)
C34	0.071 (3)	0.0235 (18)	0.047 (2)	−0.0011 (17)	0.007 (2)	−0.0012 (18)
C35	0.050 (3)	0.033 (2)	0.040 (2)	0.0016 (16)	−0.0026 (19)	−0.0037 (17)
C36	0.074 (3)	0.0215 (18)	0.041 (2)	0.0029 (17)	0.005 (2)	−0.0009 (17)
C37	0.070 (3)	0.0175 (16)	0.041 (2)	−0.0042 (17)	−0.002 (2)	−0.0005 (16)

### Geometric parameters (Å, º)

**Table:**

O1—C34	1.219 (5)	C6—C7	1.426 (5)
O1W—H1WA	0.8199	C6—H6A	0.9300
O1W—H1WB	0.8200	C7—C8	1.400 (6)
O2—C34	1.296 (5)	C7—C11	1.413 (5)
O2—H2	0.8200	C8—C9	1.356 (5)
O3—C35	1.285 (4)	C8—H8A	0.9300
O4—C35	1.228 (5)	C9—C10	1.410 (5)
O5—C36	1.307 (5)	C9—H9A	0.9300
O5—H5	0.8200	C10—C14	1.498 (5)
O6—C36	1.186 (5)	C11—C12	1.436 (5)
O7—C37	1.208 (5)	C13—H13A	0.9600
O8—C37	1.321 (5)	C13—H13B	0.9600
O8—H8	0.8200	C13—H13C	0.9600
N1—C1	1.341 (5)	C14—H14A	0.9600
N1—C12	1.369 (5)	C14—H14B	0.9600
N1—H1A	0.8600	C14—H14C	0.9600
N2—C10	1.331 (4)	C28—C33	1.392 (5)
N2—C11	1.354 (5)	C28—C29	1.409 (5)
C1—C2	1.401 (6)	C28—C34	1.521 (5)
C1—C13	1.494 (6)	C29—C30	1.403 (5)
C2—C3	1.359 (6)	C29—C35	1.530 (6)
C2—H2B	0.9300	C30—C31	1.388 (5)
C3—C4	1.408 (6)	C30—H30A	0.9300
C3—H3A	0.9300	C31—C32	1.402 (5)
C4—C12	1.400 (5)	C31—C36	1.498 (5)
C4—C5	1.429 (6)	C32—C33	1.384 (5)
C5—C6	1.337 (6)	C32—C37	1.497 (5)
C5—H5A	0.9300	C33—H33A	0.9300
			
H1WA—O1W—H1WB	110.5	C4—C12—C11	120.7 (4)
C34—O2—H2	109.5	C1—C13—H13A	109.5
C36—O5—H5	109.5	C1—C13—H13B	109.5
C37—O8—H8	109.5	H13A—C13—H13B	109.5
C1—N1—C12	123.7 (3)	C1—C13—H13C	109.5
C1—N1—H1A	118.2	H13A—C13—H13C	109.5
C12—N1—H1A	118.2	H13B—C13—H13C	109.5
C10—N2—C11	117.8 (3)	C10—C14—H14A	109.5
N1—C1—C2	117.5 (4)	C10—C14—H14B	109.5
N1—C1—C13	118.7 (4)	H14A—C14—H14B	109.5
C2—C1—C13	123.8 (4)	C10—C14—H14C	109.5
C3—C2—C1	121.0 (4)	H14A—C14—H14C	109.5
C3—C2—H2B	119.5	H14B—C14—H14C	109.5
C1—C2—H2B	119.5	C33—C28—C29	118.3 (3)
C2—C3—C4	121.0 (4)	C33—C28—C34	113.5 (3)
C2—C3—H3A	119.5	C29—C28—C34	128.0 (3)
C4—C3—H3A	119.5	C30—C29—C28	118.0 (3)
C12—C4—C3	117.3 (4)	C30—C29—C35	113.0 (3)
C12—C4—C5	119.2 (4)	C28—C29—C35	129.0 (3)
C3—C4—C5	123.6 (4)	C31—C30—C29	122.7 (3)
C6—C5—C4	120.3 (3)	C31—C30—H30A	118.6
C6—C5—H5A	119.8	C29—C30—H30A	118.6
C4—C5—H5A	119.8	C30—C31—C32	119.4 (3)
C5—C6—C7	122.4 (4)	C30—C31—C36	120.7 (3)
C5—C6—H6A	118.8	C32—C31—C36	120.0 (3)
C7—C6—H6A	118.8	C33—C32—C31	117.8 (3)
C8—C7—C11	116.3 (3)	C33—C32—C37	118.4 (3)
C8—C7—C6	124.7 (4)	C31—C32—C37	123.7 (3)
C11—C7—C6	119.0 (4)	C32—C33—C28	123.9 (3)
C9—C8—C7	119.8 (4)	C32—C33—H33A	118.1
C9—C8—H8A	120.1	C28—C33—H33A	118.1
C7—C8—H8A	120.1	O1—C34—O2	120.0 (3)
C8—C9—C10	120.4 (4)	O1—C34—C28	119.6 (3)
C8—C9—H9A	119.8	O2—C34—C28	120.4 (3)
C10—C9—H9A	119.8	O4—C35—O3	122.7 (4)
N2—C10—C9	121.6 (3)	O4—C35—C29	118.5 (3)
N2—C10—C14	117.6 (3)	O3—C35—C29	118.7 (3)
C9—C10—C14	120.8 (3)	O6—C36—O5	123.0 (3)
N2—C11—C7	124.1 (3)	O6—C36—C31	123.4 (3)
N2—C11—C12	117.6 (3)	O5—C36—C31	113.6 (3)
C7—C11—C12	118.4 (3)	O7—C37—O8	120.2 (4)
N1—C12—C4	119.6 (3)	O7—C37—C32	123.1 (4)
N1—C12—C11	119.8 (3)	O8—C37—C32	116.3 (4)
			
C12—N1—C1—C2	−0.2 (6)	C7—C11—C12—C4	2.5 (6)
C12—N1—C1—C13	−179.6 (4)	C33—C28—C29—C30	0.1 (6)
N1—C1—C2—C3	0.2 (7)	C34—C28—C29—C30	−175.6 (4)
C13—C1—C2—C3	179.6 (4)	C33—C28—C29—C35	−179.5 (4)
C1—C2—C3—C4	−0.2 (7)	C34—C28—C29—C35	4.8 (7)
C2—C3—C4—C12	0.1 (6)	C28—C29—C30—C31	0.0 (6)
C2—C3—C4—C5	−179.2 (4)	C35—C29—C30—C31	179.7 (4)
C12—C4—C5—C6	−1.6 (6)	C29—C30—C31—C32	0.0 (6)
C3—C4—C5—C6	177.7 (4)	C29—C30—C31—C36	178.6 (4)
C4—C5—C6—C7	1.7 (7)	C30—C31—C32—C33	−0.1 (6)
C5—C6—C7—C8	179.6 (4)	C36—C31—C32—C33	−178.7 (4)
C5—C6—C7—C11	0.4 (6)	C30—C31—C32—C37	176.7 (4)
C11—C7—C8—C9	1.9 (6)	C36—C31—C32—C37	−2.0 (6)
C6—C7—C8—C9	−177.3 (4)	C31—C32—C33—C28	0.2 (6)
C7—C8—C9—C10	−1.1 (6)	C37—C32—C33—C28	−176.7 (4)
C11—N2—C10—C9	0.7 (6)	C29—C28—C33—C32	−0.2 (6)
C11—N2—C10—C14	−178.3 (3)	C34—C28—C33—C32	176.1 (4)
C8—C9—C10—N2	−0.3 (6)	C33—C28—C34—O1	−8.9 (6)
C8—C9—C10—C14	178.7 (4)	C29—C28—C34—O1	167.0 (4)
C10—N2—C11—C7	0.3 (6)	C33—C28—C34—O2	170.5 (4)
C10—N2—C11—C12	−179.6 (3)	C29—C28—C34—O2	−13.6 (7)
C8—C7—C11—N2	−1.6 (6)	C30—C29—C35—O4	8.2 (5)
C6—C7—C11—N2	177.7 (3)	C28—C29—C35—O4	−172.2 (4)
C8—C7—C11—C12	178.3 (3)	C30—C29—C35—O3	−168.0 (3)
C6—C7—C11—C12	−2.5 (6)	C28—C29—C35—O3	11.6 (6)
C1—N1—C12—C4	0.1 (6)	C30—C31—C36—O6	175.9 (5)
C1—N1—C12—C11	180.0 (3)	C32—C31—C36—O6	−5.4 (7)
C3—C4—C12—N1	−0.1 (6)	C30—C31—C36—O5	−3.5 (6)
C5—C4—C12—N1	179.3 (4)	C32—C31—C36—O5	175.2 (4)
C3—C4—C12—C11	−179.9 (4)	C33—C32—C37—O7	−77.6 (5)
C5—C4—C12—C11	−0.6 (6)	C31—C32—C37—O7	105.7 (5)
N2—C11—C12—N1	2.6 (5)	C33—C32—C37—O8	95.8 (4)
C7—C11—C12—N1	−177.3 (3)	C31—C32—C37—O8	−81.0 (5)
N2—C11—C12—C4	−177.6 (3)		

### Hydrogen-bond geometry (Å, º)

**Table:**

*D*—H···*A*	*D*—H	H···*A*	*D*···*A*	*D*—H···*A*
O2—H2···O3	0.82	1.58	2.395 (4)	171
O5—H5···O1^i^	0.82	1.86	2.671 (3)	172
O8—H8···O3^ii^	0.82	1.82	2.645 (4)	178
N1—H1*A*···O1*W*^iii^	0.86	1.92	2.738 (4)	160
O1*W*—H1*WA*···O4^ii^	0.82	1.92	2.735 (4)	171
O1*W*—H1*WB*···O7^iv^	0.82	2.11	2.873 (4)	155

Symmetry codes: (i) −*x*+1/2, *y*+1/2, *z*; (ii) *x*+1/2, −*y*+1/2, −*z*; (iii) *x*−1/2, *y*, −*z*+1/2; (iv) *x*+1, *y*, *z*.
